# Obituary

**Published:** 1880-06

**Authors:** 


### OBITUARY.

Died, in Ashtabula, Ohio, on Friday, June 4th, 1880, of hemorrhage, Dr. W. L. Wallace, widely known and much respected member and practitioner of the dental profession. Dr. Wallace graduated at the Ohio College of Dental Surgery, in the class of 1871. He was not a man of aggressive disposition, was rather retiring in manner, but earnest and progressive in all that he undertook. He was held in high esteem by all who knew him.

---

Died on the 19th of April, at Carrolton, Ky., Dr. J. M. Floore, Jr., aged 26 years. Dr. Floore was one of the rising young men of the profession in Kentucky. He began practice in Carrolton in 1877, and had already made a reputation of which any one might be proud. He made friends of all with whom he came in contact, and enjoyed universal esteem and confidence.

Such men cannot be well spared from the profession, nor indeed from general society.

His family will have the sincere sympathy of all who knew him.

